# Lipocalin 24p3 Induction in Colitis Adversely Affects Inflammation and Contributes to Mortality

**DOI:** 10.3389/fimmu.2019.00812

**Published:** 2019-04-17

**Authors:** Zhuoming Liu, Fabio Cominelli, Luca Di Martino, Ruifu Liu, Neha Devireddy, Lax R. Devireddy, David N. Wald

**Affiliations:** ^1^Department of Pathology, Case Western Reserve University, Cleveland, OH, United States; ^2^Department of Medicine, Case Western Reserve University, Cleveland, OH, United States; ^3^Beaumont High School, Shaker Heights, OH, United States; ^4^Department of Pathology, University Hospitals Cleveland Medical Center, Cleveland, OH, United States

**Keywords:** 24p3, colitis, iron metabolism, PDGF, inflammatory bowel disease

## Abstract

Recognition of microorganism associated molecular patterns by epithelial cells elicits signaling cascades resulting in the production of host defense proteins. Lipocalin 24p3 is purported to be one such protein. 24p3 binds prokaryotic and eukaryotic siderophores and by sequestering iron laden bacterial siderophores it was believed to restrict bacterial replication. As such mice deficient for 24p3 are susceptible to systemic infections. However, it is not clear whether deficiency of 24p3 on the gut mucosa contributes to inflammation. In line with 24p3's function as a bacteriostat, it would be reasonable to assume that deficiencies in the control of intestinal flora from 24p3 absence play a role in inflammatory intestinal diseases. Surprisingly, we show 24p3 is a contributor of inflammation and 24p3 deficiency protects mice from dextran sodium sulfate (DSS)-induced colitis. 24p3 was found to be a negative regulator of platelet-derived growth factor (PDGF), which helps maintain the integrity of the gut mucosa. Neutralization of PDGF-BB abrogated resistance of 24p3 null mice to DSS confirming the direct link between 24p3 and PDGF-BB. Finally, iron handling in wild-type and 24p3-null mice upon DSS treatment also differed. In summary, differential iron levels and enhanced expression of PDGF-BB in 24p3 null mice confers resistance to DSS.

## Introduction

The mammalian gastrointestinal tract harbors more than a trillion microbes, which are collectively referred to as microbiota (1). The host provides protection and nutrients to microbiota and microbiota aid in digestion. Despite this mutually beneficial aspect of colonization of microbiota on the intestinal surface, the proximity of microbes and their associated antigens to the epithelia of host intestine poses a major challenge to the host to mitigate the potential for opportunistic invasion of intestinal epithelial cells [IECs; (2, 3)]. In healthy individuals the intestinal epithelial barrier protects underlying mucosal tissues from commensal gut microbes. In susceptible individuals or after exposure to dextran sodium sulfate (DSS) the epithelial barrier is compromised allowing commensal microbial flora to invade the gut (4, 5). Macrophages residing in intraepithelial spaces and intestinal epithelial cells sample microbial antigens and induce a regulatory immune response or release proteins with antimicrobial properties (6).

Lipocalin 24p3, a member of the lipocalin family of carrier proteins, is one such protein that is purported to regulate intestinal microbiota (7–9). Originally isolated from secondary granules of neutrophils (10, 11), 24p3 is also found in mucus producing epithelial cells lining respiratory (12–14) and intestinal tracts (15), in liver in response to acute phase or sepsis (16), and in macrophages consequent to stimulation by TLRs (17). 24p3 is an iron binding protein though it lacks the ability to bind iron directly rather iron binding is mediated by a siderophore (18). Thus, by sequestering iron laden siderophores 24p3 acts as a bacteriostat. Therefore, mice rendered deficient for 24p3 are reported to be sensitive to infectious agents whose siderophores are a target for 24p3 (12–14, 17, 19, 20). However, all these studies utilized *24p3* deficient mice made on the C57BL/6J genetic background, which carry a deletion in group II phospholipase A2 gene (PLA2), an antibacterial acute phase protein (21, 22). This this gene deletion may explain why these mice are sensitive to even the LD_30_ of the *E. coli* strain 25922 (23). Enforced expression of *pla2* reverses this sensitivity (23). Thus, it appears that the presence or absence of PLA2 determines the sensitivity of C57BL/6J mice to *E.coli* regardless of the status of 24p3. Studies have not been previously conducted to assess the contribution of 24p3 to innate immunity in C57BL/6J mice rendered positive for PLA2.

Hepcidin, a hormone secreted by the liver regulates systemic iron by altering the expression of ferroportin on the plasma membrane (24). Ferroportin expressed in duodenal enterocytes or splenic macrophages exports iron into the blood stream absorbed from dietary sources or via recycled red blood cells, respectively (24). Thus, by regulating ferroportin, hepcidin controls systemic iron levels to maintain iron homeostasis (24). However, under inflammatory conditions, hepcidin levels are elevated, which then leads to hypoferremia. In addition to hepcidin, other proteins that regulate iron metabolism such as ferritin are also altered in inflammation (25). Iron levels are also regulated via hepcidin-independent mechanisms in inflammation (26). Even though 24p3 is implicated in iron transport its role in iron metabolism of inflammation is not clear. Equally intriguing is the relationship between 24p3 and other systemic regulators of iron homeostasis. To this end, it was shown that 24p3 levels were significantly upregulated in hepcidin-deficient mice suggesting that 24p3-mediated iron trafficking may be an alternative pathway for hypoferremia in these mice (27).

24p3 has a dual role in inflammation: pro-inflammatory or anti-inflammatory depending on conditions (8, 13, 28). Several studies found an elevation in 24p3 levels in IBD specimens and the importance of such elevated levels of 24p3 is unknown (15, 29–31). However, in line with the general notion that 24p3 is a bacteriostat (18) it is reasonable assumption that constitutive secretion of 24p3 by gut epithelium is important for immune surveillance and a defect because of 24p3 deficiency may play a role in inflammatory diseases of intestines. Contrary to this prediction, we found that 24p3 is a contributor of inflammation and 24p3 deficiency protects mice from DSS-induced colitis. In addition, we also found that 24p3 negatively regulates expression of platelet-derived growth factor-BB (PDGF-BB), which is critical for maintaining the mucosal integrity. Neutralization of PDGF-BB levels by antibody administration eroded resistance of *24p3*^−/−^ mice to DSS. Finally, iron handling in *24p3*^+/+^ and *24p3*^−/−^ mice upon DSS treatment also differed. In summary, differential iron levels and enhanced expression of PDGF-BB in *24p3*^−/−^ mice confers resistance to DSS.

## Materials and Methods

### Mice

Derivation of *24p3*^−/−^ mice is described elsewhere (32). Mice were housed in a pathogen-free facility and all animal protocols were approved by the Institutional Animal Care and Use Committee of Case Western Reserve University. *24p3*^−/−^ mice were maintained on a C57BL/6J genetic background. All experiments were performed using age- and gender-matched *24p3*^+/+^ mice.

### Experimental Colitis

Mice were administered with 4% (w/v) DSS (average molecular weight 40,000 g/mol; PanReac AppliChem, Maryland Heights, MO) dissolved in sterile, distilled water *ad libitum* for 2 weeks. Fresh DSS solution was replenished every third day.

### Determination of Clinical Scores

Mice were examined daily for weight loss and evidence of colitis by examining stool consistency and the presence of occult blood. The baseline clinical score was determined on day 1 post DSS administration.

### Endoscopy

Mice were anesthetized with 4% isoflurane prior to endoscopy. Colonoscopy was performed on the 7th day of post DSS treatment and inflammation was evaluated by a previously validated endoscopic scoring system (33), which incorporates 4 different parameters to assess colonic inflammation: perianal findings (diarrhea, bloody feces or rectal prolapse), wall transparency (ability to observe colonic mucosal blood vessels), intestinal bleeding (spontaneous or procedurally induced by endoscope due to mucosal friability), and focal lesions (edema, erosions and ulcers). Colonoscopy was performed as described in Di Martino et al. (34) using a flexible digital ureteroscope (URF-V, Olympus America, Center Valley, PA) with an 8.5 Fr (2.8 mm) tapered-tip design and a motion range of 180° in an up angle and 275° in a down angle. The endoscope system includes a video system center (Olympus America), a xenon light source (Olympus America) and a video recorder (Medi Capture, Plymouth Meeting, PA). Sub scores for each parameter ranging from 0 (normal colonoscopy) to 3 (maximum severity of colonic changes) were used to evaluate colonic inflammation. The sum of these sub scores was used to define colonic health as follows: healthy (0–1), mild colitis (2–4), and moderate colitis (5–7).

### Determination of Blood Cell Numbers

Whole blood from naïve as well as DSS administered *24p3*^+/+^ and *24p3*^−/−^ mice was collected by tail vein puncture and blood cell counts were determined in an automated blood counter (IDEXX, Westbrook, ME).

### Assessment of Serum 24p3 Levels

24p3 levels in naïve as well as DSS administered mice were measured using a commercial ELISA kit from (R& D systems, Minneapolis, MN) as per the manufacturer's instructions.

### Cytokine Measurements

Serum was collected from blood collected by cardiac puncture at indicated time points. Mouse cytokines and chemokines in sera were measured using cytokine 9-Plex discovery (Eve technologies, Calgary, Alberta, Canada).

### RNA Isolation and Gene Expression Analysis

Total RNA was isolated from liver samples using the Trizol method (Invitrogen). DNase I (Promega) treated RNA was reverse transcribed using Superscript III RT from Invitrogen as per the manufacturer's recommendations. The resulting cDNAs were used for real time PCR analysis using SYBR Green master mix (Promega, Madison, WI) following the manufacturer's recommendations. The fold-change was calculated using the ΔΔCT method.

### Iron Measurements

Iron levels in sera and tissue samples were measured as described (35).

### PDGF-BB Neutralization in DSS-Induced Colitis

DSS administered mice were injected daily with 10 μg of anti PDGF-BB antibody (EMD Millipore, Burlington, MA) via intraperitoneally. Survival rate was assessed 2 weeks after completion of the experiment.

### Pyrosequencing

Fecal samples from naïve and DSS administered mice were collected and DNA was extracted using a commercial kit from Qiagen as per manufacturer's instructions. DNA concentrations were adjusted, standardized and subjected to PCR amplification using a kit from Illumina using primers specific for 16s rRNA gene. Pooled libraries were then assessed for quality and sent for sequencing at Texas A & M university. Sequence analysis was performed in the laboratory of Dr. Jan Suchodolski at Texas A & M as per established procedures (36).

### Statistical Analysis

Data are presented as mean ± SD. Error bars shown in figures represent SD. Statistical analyses were performed by one-way analysis of variance and the Tukey HSD (honestly significant difference) test was performed for multiple comparisons using SAS/STAT software. For a two-way comparison, Students *t*-test was used with Welch's correction. *P* < 0.05 were considered statistically significant.

### Study Approval

The Case Western Reserve University IACUC committee approved all of the animal studies performed.

## Results

### Damage to Colonic Epithelium Induces 24p3

**Figure 1:**
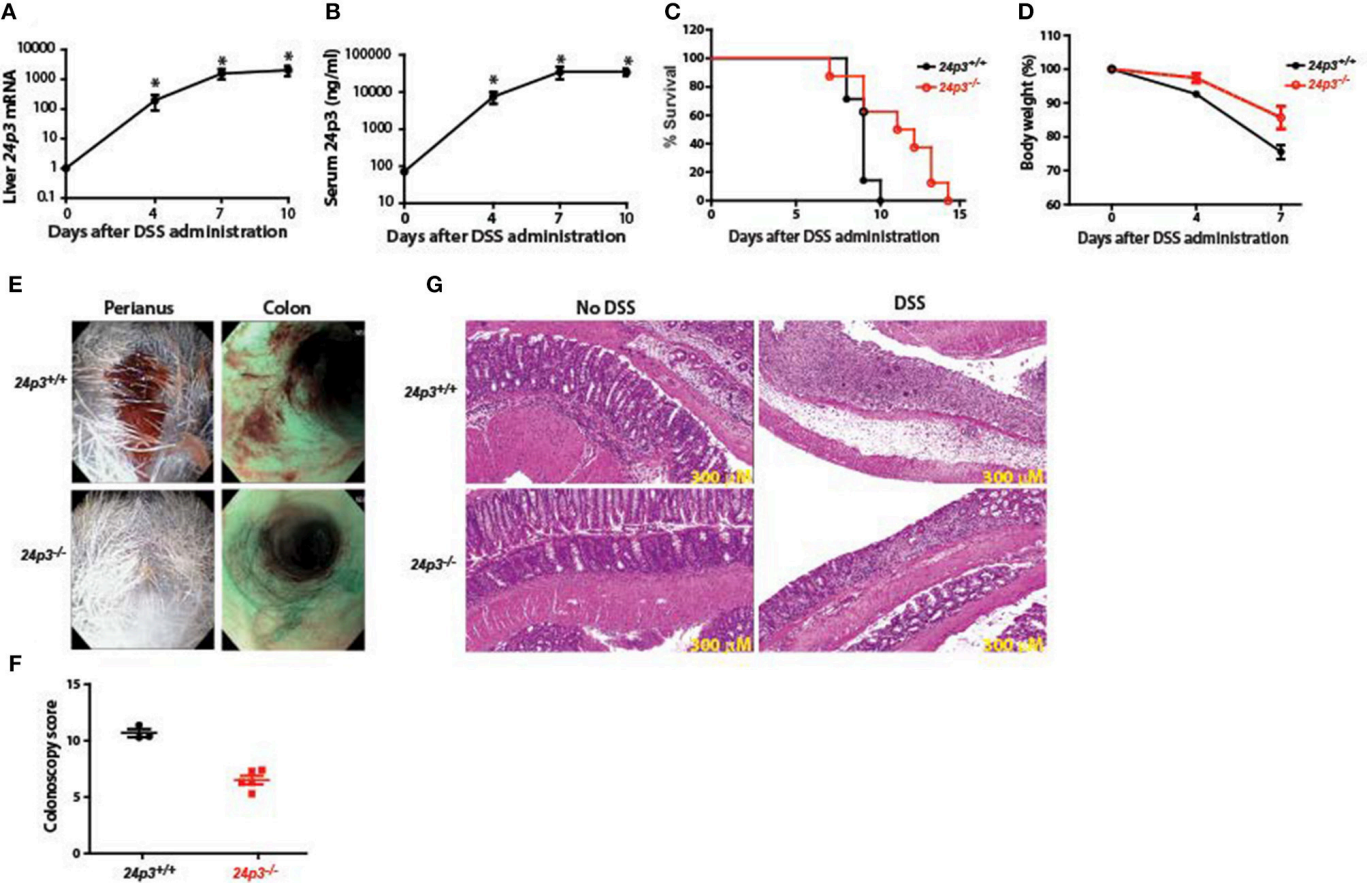
*24p3*^−/−^ mice are resistant to DSS-induced colitis. **(A,B)** DSS treatment induces 24p3 as judged by real time PCR analysis **(A)** and serum ELISA **(B)**. ^*^p ≤ 0.05. **(C)** Kaplan-Meier analysis of survival rate of *24p3*^+/+^ and *24p3*^−/−^ mice. Mice were administered with 4% DSS in drinking water and survival was monitored for 2 weeks. *p* ≤ 0.05 for pair wise comparison. **(D)** Body weight loss of *24p3*^+/+^ and *24p3*^−/−^ mice after DSS administration. Data is expressed as the percentage of initial body weight. ^*^p ≤ 0.05. **(E)** Perianal images and distal colon endoscopy images of mice at day 7 post exposure to DSS. Both images show severe bleeding and extensive mucosal inflammation in *24p3*^+/+^ mice and mild bleeding and inflammation in *24p3*^−/−^ mice. **(F)** Endoscopy scores of *24p3*^+/+^ and *24p3*^−/−^ mice at day 7 post exposure to DSS. **(G)** Representative H&E-stained sections of colons from naïve and DSS administered *24p3*^+/+^ and *24p3*^−/−^ mice sampled on day 7.

Oral administration of DSS is toxic to the colonic epithelium and triggers a local inflammatory reaction by exposing the lamina propria of the colon to resident gut flora (4, 5). These invaders also spread systemically via a hematogenous route thus triggering a wide spread acute phase-like reaction. In accordance with previous findings that 24p3 is an acute phase protein (16), we found that 24p3 levels were increased by >1,000-fold at the RNA level and >200-fold at the protein level, respectively in DSS-treated *24p3*^+/+^ mice (Figures 1A,B). Such an acute increase in 24p3 levels upon DSS treatment suggests that it may play an important role in the inflammatory response to DSS.

### 24p3 Null Mice Are Resistant to Dextran Sodium Sulfate (DSS)-Induced Colitis

DSS-induced Ulcerative Colitis (UC)-like condition is a widely accepted model to uncover phenotypes in gene knockout mice (37, 38). Age- and sex-matched *24p3*^+/+^ and *24p3*^−/−^ mice were exposed to 4% DSS in their drinking water to induce a UC-like condition. We found that DSS-administered *24p3*^+/+^ mice rapidly lost weight and succumbed to colitis with an onset of death as early as 7 days post DSS exposure with nearly all mice dying within 10 days after administration of DSS (Figures 1C,D). In contrast, only 40% of DSS-administered *24p3*^−/−^ mice died on day 10 compared to the *24p3*^+/+^ cohort suggesting that these mice are relatively resistant to DSS-induced colitis (Figure 1C). Eventually, all DSS-administered *24p3*^−/−^ mice succumbed to DSS-induced colitis at day 15 (Figure 1C). In addition, we also found that DSS treated *24p3*^+/+^ mice rapidly lost weight whereas the loss of body weight was more gradual in *24p3*^−/−^ mice (Figure 1D). The loss of body weight is concomitant with survival in both groups of DSS treated mice. Differences in rectal bleeding were also apparent between the two groups. Gross examination of colon by endoscopy revealed hemorrhagic diarrhea in DSS treated *24p3*^+/+^ mice whereas the severity of bleeding was significantly lower in DSS treated *24p3*^−/−^ mice (Figure 1E). A quantitative assessment of colonoscopy findings also yielded significant differences between *24p3*^+/+^ and *24p3*^−/−^ mice upon DSS exposure (Figure 1F).

These clinical assessments were validated histologically using representative colon sections. As expected, we observed marked histopathological changes in hematoxylin & eosin (H&E)-stained colons from DSS-administered *24p3*^+/+^ mice characterized by crypt loss, infiltrating leukocytes, ulceration, and submucosal edema (Figure 1G). In contrast, colon sections from DSS-administered 24p3^−/−^ displayed mild to moderate inflammation (Figure 1G). Consistent with the absence of disease in naïve mice that were not administered DSS, no signs of inflammation or tissue damage were observed in their colons (Figure 1G). Semiquantitative scoring of these histological sections confirmed that colitis severity in DSS-administered *24p3*^+/+^ mice was higher than DSS-administered *24p3*^−/−^ mice (data not shown). In summary, DSS-administered *24p3*^+/+^ mice bore all the hallmarks of colitis and succumbed to DSS treatment while *24p3*^−/−^ mice exhibited a reduced sensitivity to DSS-induced colitis. These results demonstrate that 24p3 plays a role in the progression of DSS-induced colitis.

### DSS Treatment Does Not Alter Leukocyte Numbers in 24p3^−/−^ Mice

**Figure 2:**
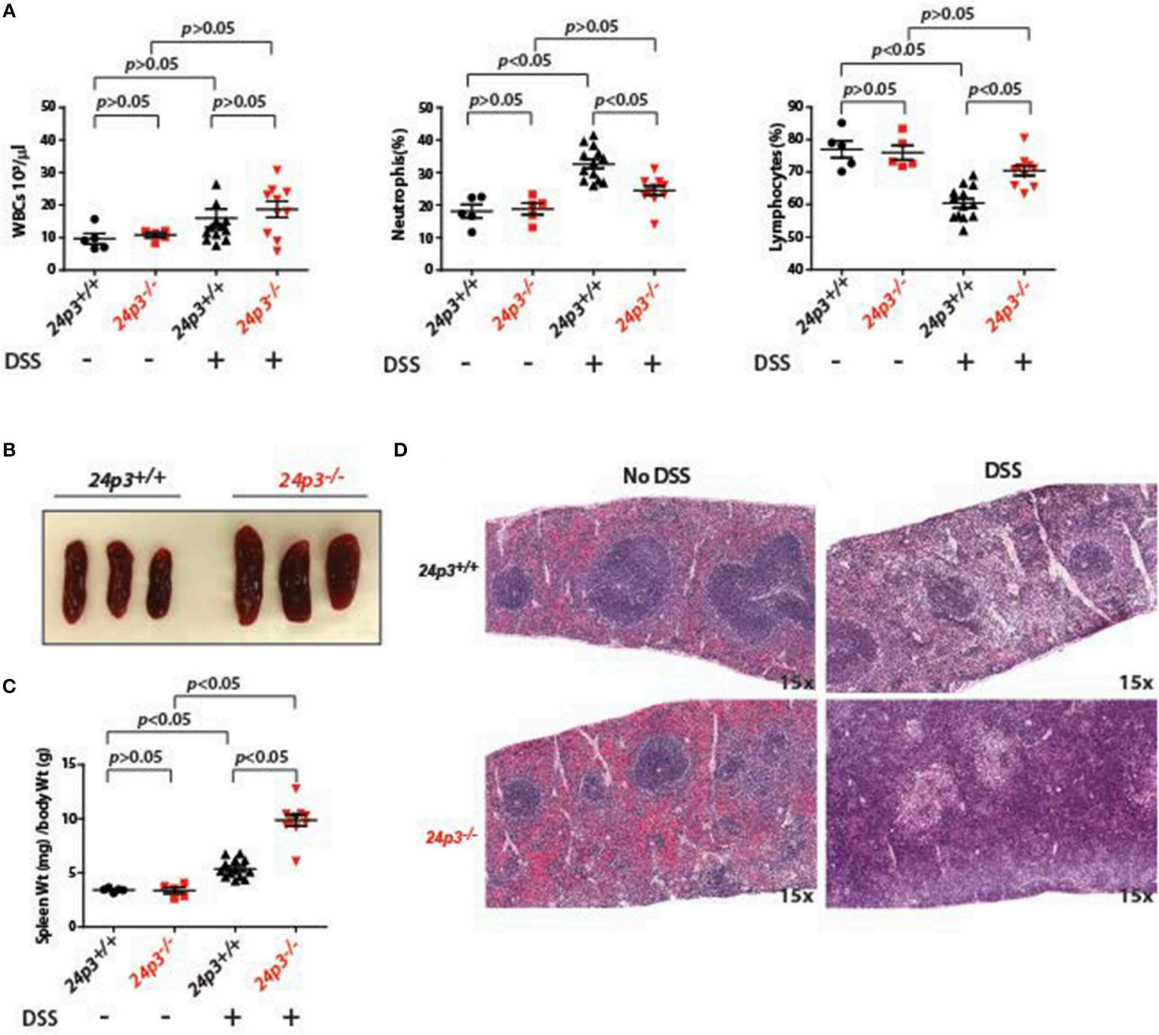
Splenomegaly in DSS administered *24p3*^−/−^ mice. **(A)** Total leukocyte, neutrophil and lymphocyte counts in naïve and DSS administered *24p3*^+/+^ and *24p3*^−/−^ mice. **(B)** Gross images of spleens of DSS administered *24p3*^+/+^ and *24p3*^−/−^ mice. **(C)** Organ/body weight ratios naïve and DSS administered *24p3*^+/+^ and *24p3*^−/−^ mice. **(D)** Representative H&E-stained sections of spleens from naïve and DSS administered *24p3*^+/+^ and *24p3*^−/−^ mice.

24p3 is a regulator of immune cells (35). To test whether the observed resistance of *24p3*^−/−^ mice to DSS was indeed conferred by a change in systemic inflammatory response, we performed differential blood cell counts at day 7 post DSS exposure in both groups of mice. There was no significant difference in baseline circulating leukocyte counts in naïve mice as well as in DSS administered mice of both genotypes (Figure 2A). However, upon DSS exposure there was an increase in neutrophil counts in *24p3*^+/+^ mice but not in *24p3*^−/−^ mice (Figure 2A). In contrast, lymphocyte counts were decreased in DSS administered *24p3*^+/+^ mice compared to naïve *24p3*^+/+^ mice (Figure 2A).

Interestingly, exacerbated inflammatory response in DSS-administered *24p3*^+/+^ mice is not associated with splenomegaly. Based on the organ/body weight ratio, naïve as well as DSS-administered *24p3*^+/+^ mice displayed no changes in spleen (Figures 2A,B). However, we observed marked splenomegaly in DSS exposed *24p3*^−/−^ mice (Figure 2B). Based on the organ/body weight ratio, the DSS-administered *24p3*^−/−^ mice had ~2.2-fold larger spleens than DSS-administered *24p3*^+/+^ mice (Figure 2C). We next examined the histological sections of spleens of naïve as well as DSS administered *24p3*^+/+^ mice and *24p3*^−/−^ mice to gain insight into the observed splenomegaly. While there are no significant changes in the splenic architecture of the *24p3*^+/+^ mice regardless of treatment, we found that there was significant follicle destruction and infiltration of inflammatory cells in spleens of *24p3*^−/−^ mice treated with DSS (Figure 2D).

### Microbiota Is Not Altered in 24p3^−/−^ Mice Treated With DSS

**Figure 3:**
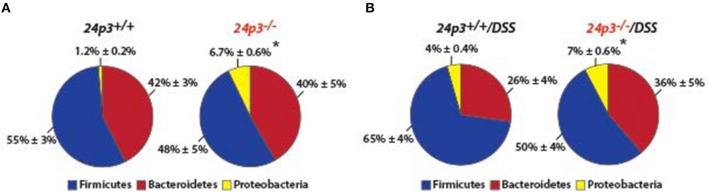
Gut microbiota is unaltered upon DSS administration in *24p3*^−/−^ mice. **(A,B)** Gut microbiome analysis of naïve and DSS administered *24p3*^+/+^ and *24p3*^−/−^ mice. Relative abundance of major bacterial phyla is shown. The asterisk indicates the significant difference in the percentage of Proteobacteria between the wild-type and knockout mice by Student's *t*-test.

24p3 is a bacteriostat therefore it was purported that 24p3 may be required to mount a proper response to prevent overgrowth of commensal microflora in the lumen of the colon in response to intestinal inflammation because of DSS treatment (7). To examine whether resident gut flora were altered in the absence of 24p3 in naïve as well as in DSS administered mice, we performed 16 s rRNA sequencing of fecal samples. We found significantly more bacteria belonging to phylum *Proteobacteria* in naïve *24p3*^−/−^ mice as compared to naive *24p3*^+/+^ mice (Figures 3A,B). However, DSS treatment did not cause an increase in these bacteria in *24p3*^−/−^ mice (Figure 3B). *Bacteroidetes* phylum, was rather decreased upon exposure to DSS in *24p3*^+/+^ mice but not in DSS-administered *24p3*^−/−^ mice (Figures 3A,B). *Firmicutes* were increased in DSS-administered *24p3*^+/+^ mice but not in DSS-administered *24p3*^−/−^ mice (Figures 3A,B). In summary, *24p3*^+/+^ mice but not *24p3*^−/−^ mice displayed significant changes in composition of bacteria phyla in response to DSS treatment.

### Altered Cytokine Expression in DSS Administered 24p3^−/−^ Mice

**Figure 4:**
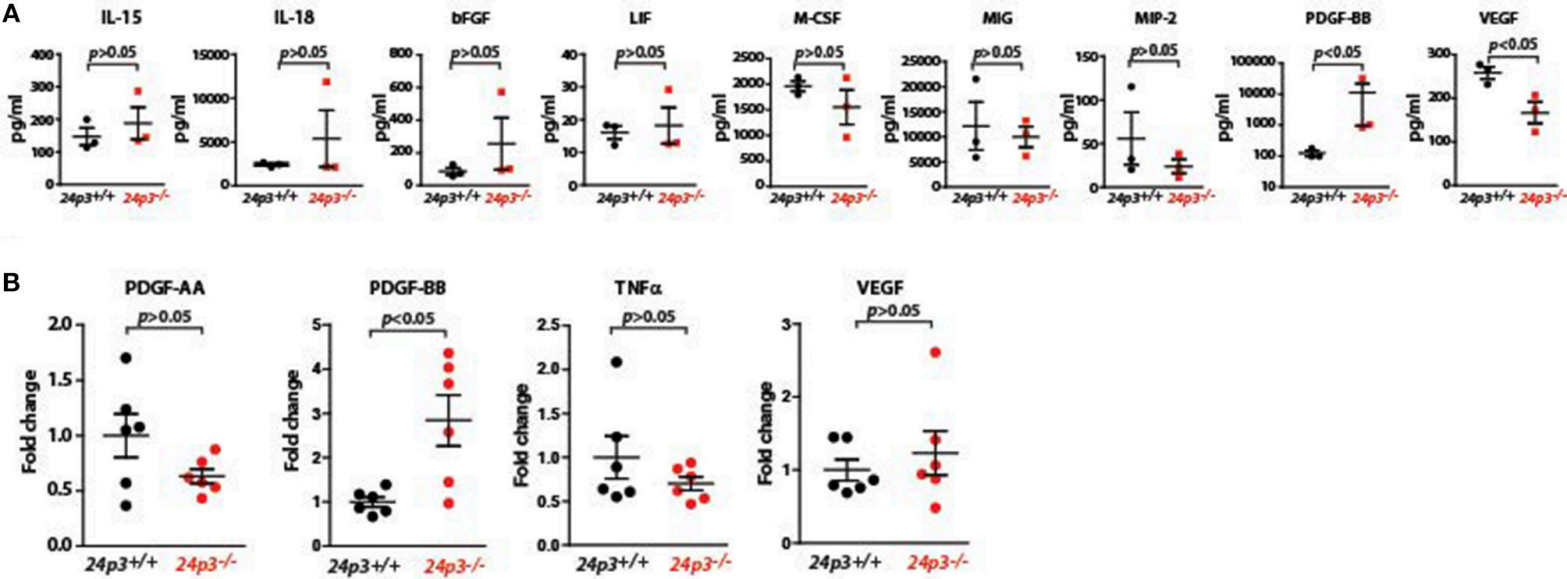
Analysis of cytokines in DSS administered *24p3*^−/−^ mice **(A)** Assessment of cytokines in DSS administered *24p3*^+/+^ and *24p3*^−/−^ mice by a multiplex assay. **(B)** Assessment of *pdgf-aa* and *pdgf-bb, tnf*α and *vegf* in colonic tissue of naïve and DSS administered *24p3*^+/+^ and *24p3*^−/−^ mice by real time PCR.

Systemic dissemination of bacteria and their components following the loss of epithelial barrier may result in profound changes in cytokine and chemokine responses. To examine this, we measured a variety of cytokines (IL-15, IL-18, bFGF, PDGF-BB, VEGF, TNFα, and LIF) and chemokines (M-CSF, MIP-2, MIG) in sera of DSS administered mice of both genotypes. Among all the components that were analyzed, the amounts of PDGF-BB levels were significantly higher in serum of *24p3*^−/−^*mice* relative to DSS administered *24p3*^+/+^ mice (Figure 4A). In contrast, levels of chemokines and pro-inflammatory cytokines were unaltered despite DSS treatment in *24p3*^−/−^ *mice* (Figure 4A).

Cytokines locally expressed in the colonic mucosa are differentially expressed following DSS treatment. Therefore, we also evaluated colonic cytokine production in *24p3*^+/+^ and *24p3*^−/−^ mice challenged with DSS by assessing transcript levels of *pdgf-aa, pdgf-bb, tnf*α, and *vegf* (Figure 4B). In agreement with the increased serum PDGF-BB levels, we found that transcript levels of *pdgf-bb* were also higher in colonic tissue of *24p3*^−/−^ mice administered with DSS compared to DSS administered *24p3*^+/+^ mice (Figure 4B). Expression levels of *pdgf-aa, tnf*α, and *vegf* remain unaltered (Figure 4B). In summary, we found differentially altered expression of PDGF-BB in response to DSS treatment in *24p3*^−/−^ mice.

### Altered Iron Metabolism in DSS Administered 24p3 Null Mice

**Figure 5:**
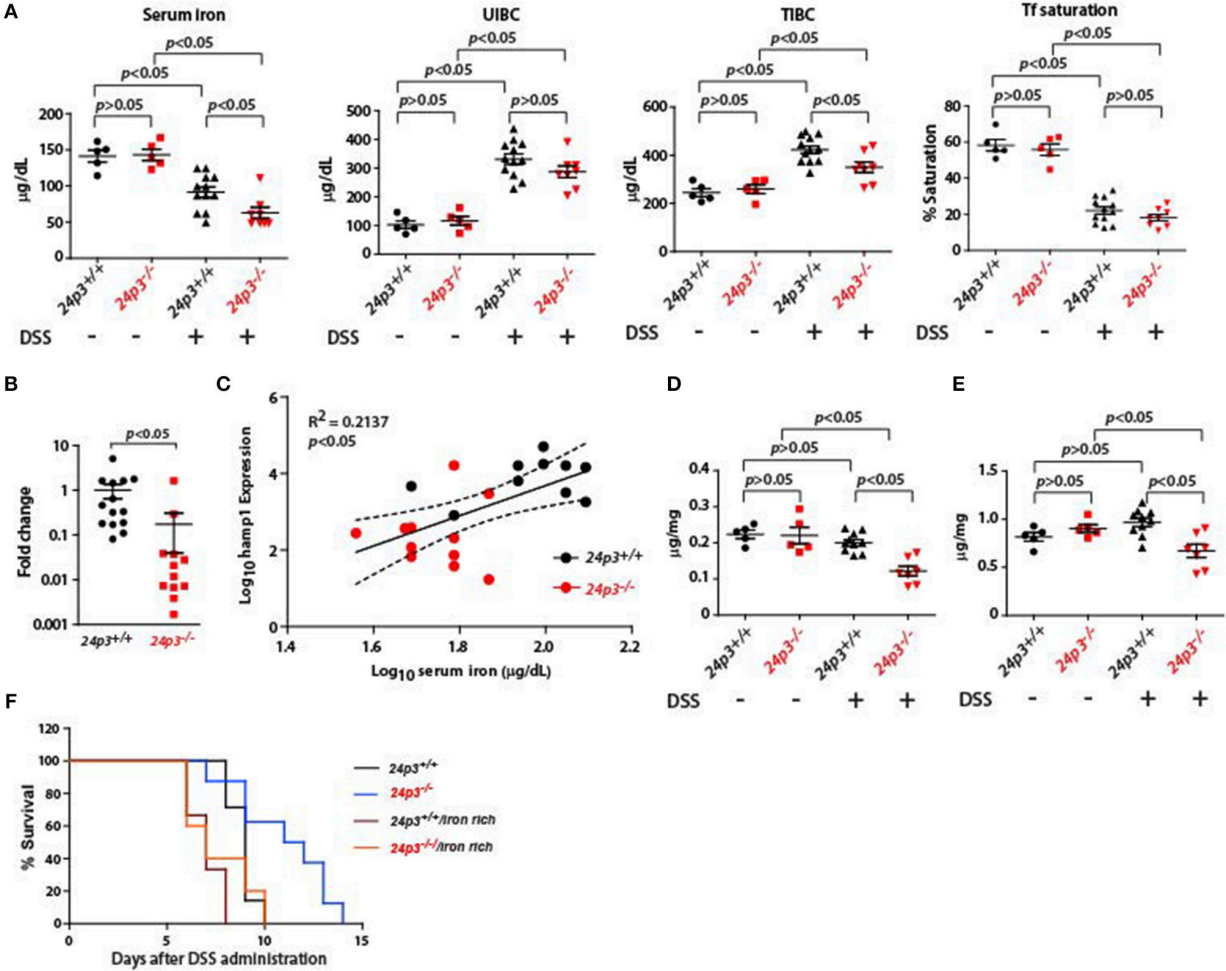
Iron measurements in naïve and DSS administered *24p3*^+/+^ and *24p3*^−/−^ mice. **(A)** Serum iron parameters in naïve and DSS administered *24p3*^+/+^ and *24p3*^−/−^ mice. **(B)** Real time PCR analysis of liver *hepcidin* expression *24p3*^+/+^ and *24p3*^−/−^ mice administered with DSS. **(C)** Regression analysis of *hepcidin* expression vs. serum iron in DSS administered *24p3*^+/+^ and *24p3*^−/−^ mice. ^*^p ≤ 0.05. **(D,E)** Liver and spleen iron measurements in naïve and DSS administered *24p3*^+/+^ and *24p3*^−/−^ mice. **(F)** Kaplan-Meier analysis of survival rate of *24p3*^+/+^ and *24p3*^−/−^ mice injected with iron dextran and subjected to DSS treatment. Survival was monitored for 2 weeks.

The hepcidin-ferroportin axis plays an important role in the regulation of iron levels in inflammation (24). Based on structural studies it was found that 24p3 is an iron chelator, but it's *in vivo* role in systemic iron metabolism is unclear. Interestingly, 24p3 levels were significantly elevated in *hepcidin* null mice suggesting that it may compensate for hepcidin loss (27). We examined the role of this axis in iron metabolism using DSS treated *24p3*^+/+^ and *24p3*^−/−^ mice. In agreement with previous studies, we found that hepcidin levels were elevated in DSS treated *24p3*^+/+^ mice [Figure 5A; (39)]. However, hepcidin levels were lower in DSS treated *24p3*^−/−^ mice (Figure 5A).

Hepcidin leads to hypoferremia and to determine whether differential levels of hepcidin in DSS treated *24p3*^+/+^ and *24p3*^−/−^ mice contributes to altered serum iron, we measured serum iron indices. We found that total serum iron was lower in DSS administered *24p3*^−/−^ mice (Figure 5B). Transferrin saturation although lower in DSS treated *24p3*^−/−^ mice but did not attain statistically significant levels (Figure 5B). Regression analysis further confirmed an inverse correlation of hepcidin and serum iron (Figure 5C). In addition, we also found that iron levels in the liver and spleen were also lower in DSS administered *24p3*^−/−^ mice (Figure 5D). It is possible that lower systemic iron levels may be one of the pathways for resistance of *24p3*^−/−^ mice to DSS. To test whether exogenous supplementation of iron reverses DSS resistance in *24p3*^−/−^ mice, we injected iron dextran into both *24p3*^+/+^ and *24p3*^−/−^ mice and subjected them to DSS treatment. As expected naïve *24p3*^−/−^ mice were resistant to DSS. However, exogenous supplementation of iron dextran resulted in an erosion of resistance to DSS in these mice (Figures 5E,F). Interestingly, exogenous administration of iron dextran also conferred enhance sensitivity to DSS administration in *24p3*^+/+^ mice (Figures 5E,F).

### Elevated Levels of PDGF-BB Confer Resistance to DSS in 24p3 Null Mice

**Figure 6:**
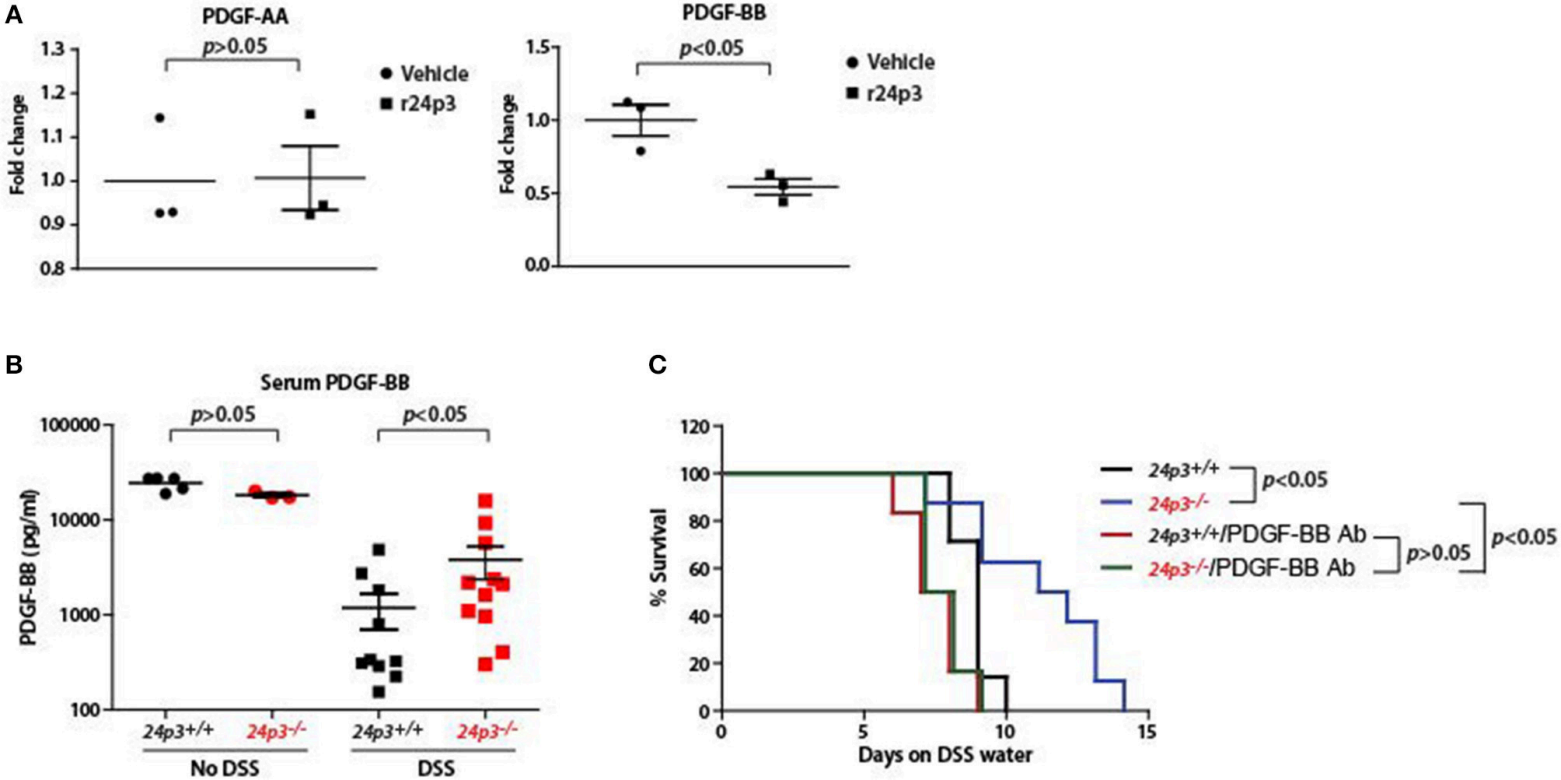
Increased PDGF-BB production in DSS administered *24p3*^−/−^ mice. **(A)** Real time PCR analysis of *pdgf-aa* and *pdgf-bb* expression in cultured colonic tissue of *24p3*^+/+^ and *24p3*^−/−^ mice administered with DSS with or without ectopic 24p3 treatment. **(B)** Assessment of serum levels of PDGF-BB in naïve as well as DSS administered *24p3*^+/+^ and *24p3*^−/−^ mice. **(C)** Kaplan-Meier analysis of survival rate of *24p3*^+/+^ and *24p3*^−/−^ mice injected with anti-PDGF-BB antibody and administering 4% DSS in drinking water. Survival was monitored for 2 weeks.

If 24p3 is a direct regulator of *pdgf-bb* then ectopic addition of 24p3 should neutralize *pdgf-bb* expression. To test this, we cultured colonic tissues from DSS-administered *24p3*^+/+^ and *24p3*^−/−^ mice and ectopically added recombinant 24p3 and assessed *pdgf-bb* transcripts (Figure 6). We detected higher transcripts of *pdgf-bb* in colonic tissue of DSS administered *24p3*^−/−^ mice (Figure 6A). However, upon addition of recombinant 24p3, we found that *pdgf-bb* levels were reduced in colonic tissue of DSS administered *24p3*^−/−^ mice (Figure 6A). To additionally confirm these results, we assed pdgf-aa levels and found their levels to remain unchanged (Figure 6A). Thus, these results show that *pdgf-bb* is negatively regulated by 24p3.

PDGF-BB is found to exhibit protective and mitogenic activities that appear to be essential for the proliferation of cells in the normal gut mucosa as well as for the repair and healing process occurring in the damaged gut mucosa (40). Finally, to directly test the contribution of PDGF-BB in the resistance of *24p3*^−/−^ mice to DSS treatment, we neutralized PDGF-BB by administering anti-PDGF-BB antibody and assessed the survival post DSS administration. In conformity with the results of Figure 4, we detected a higher level of PDGF-BB in DSS administered *24p3*^−/−^ mice (Figure 6B). However, upon injection with an anti-PDGF-BB antibody we observed enhanced sensitivity to DSS in *24p3*^−/−^ mice when compared to control uninjected *24p3*^−/−^ mice (Figure 6C). Thus, these results suggest that increased resistance of *24p3*^−/−^ mice to DSS is in part mediated by PDGF-BB and neutralization of this response abrogates the resistance.

## Discussion

We show here that *24p3*^−/−^ mice were more resistant to DSS-induced colitis. In addition, colitis-associated body weight loss, rectal bleeding, and mortality were less in *24p3*^−/−^ mice suggesting an important role for 24p3 in DSS-induced colitis. 24p3 is an acute phase protein, which is induced under inflammatory conditions. However, the role of 24p3 in inflammation is debatable. It plays both pro- and anti-inflammatory roles depending on the experimental system (7, 13, 28). For instance, it was shown that *24p3*^−/−^ mice are either sensitive or resistant to experimental colitis (7, 8). We found that *24p3*^−/−^ mice are rather resistant to DSS-induced colitis and several observations made in DSS administered *24p3*^−/−^ mice may explain the basis for this resistance: First, neutrophil response is blunted in DSS treated *24p3*^−/−^ mice—neutrophils can be both protective and detrimental depending on their numbers (41); Second, gut microbiome is not significantly altered despite DSS treatment; Third, systemic iron levels are significantly lower in DSS treated *24p3*^−/−^ mice and correction of this anomaly with extraneous iron reverses the resistance to DSS; Finally, 24p3 deficiency results in enhanced PDGF-BB expression thus contributing to repair of damaged to epithelium. Therefore, a combination of altered signaling pathways together with a lower systemic iron levels may explain, in part, the resistance of *24p3*^−/−^ mice to DSS-induced colitis. Of note, the studies here utilized mice with complete 24p3 deficiency. Future studies employing 24p3 intestinal conditional knockout mice will be of interest as both intestinal epithelial cells as well as tissue macrophages play important roles in regulating iron.

Oral administration of DSS is directly toxic to the gut and causes mucosal erosion and ulceration. Epithelial damage induces a localized repair response characterized by increased division of stem cells at the base of crypts to replace damaged enterocytes (42, 43). Cytokines such as IL-18, PDGF and bFGF are associated with repair and restitution of ulcerated epithelium by promoting proliferation or suppressing apoptosis in intestinal crypt stem cells (40). Most of the proposed functions of PDGF related to different responses to injury seen in inflammation and wound healing (40, 44). PDGF is synthesized by many cells including monocytes/macrophages. PDGF ligands signal through two related receptor tyrosine kinases, PDGFR-α and PDGFR-b. PDGFR-α is required for correct structuring of the mucosal lining of the gastrointestinal tract and mice deficient for PDGFR-α exhibit fewer villi (45). These studies show that PDGF signaling is important maintenance of mucosal lining of the gut. PDGF-BB levels are elevated in *24p3*^−/−^ mice both systemically as well as in colonic tissue suggesting that 24p3 negatively regulates expression of PDGF-BB *in vivo*. In addition, colon sections cultured in the presence of recombinant 24p3 expressed reduced levels of *pdgf* suggesting a direct connection between 24p3 and pdgf. How 24p3 regulates *pdgf* is unclear. However, it is possible that 24p3 may indirectly affect *pdgf* expression by altering iron metabolism or other signaling pathways. Altered signaling pathways in the absence of 24p3 is not unprecedented. For instance, ER signaling is down regulated in *24p3*^−/−^ mice leading to hyperlipidemia and fatty liver (46).

Global changes in microbiota have been associated with IBD in human patients with an overabundant Proteobacteria and decreased Firmicutes and Bacteriodes (47). We and others also have found that Proteobacteria were higher in naïve *24p3*^−/−^ mice suggesting that 24p3 plays a role in maintaining Proteobacteria number (7). However, Proteobacteria remain unchanged despite DSS treatment in *24p3*^−/−^ mice suggesting that 24p3 independent mechanisms keep a check on these bacteria. One connection might be that lower systemic iron levels observed in DSS treated *24p3*^−/−^ mice, which might be important for the growth and expansion of Proteobacteria especially the members of family Enterobacteriaceae. In support of this notion we found that exogeneous supplementation of iron significantly enhanced DSS-induced mortality in *24p3*^−/−^ mice.

Iron supplementation enhances DSS-induced colitis (48). We observed a lower serum iron levels in DSS treated *24p3*^−/−^ mice compared to DSS treated wt counterparts. In addition to playing a role in limiting bacterial expansion especially following DSS treatment, lower iron levels may in general affect the degree of inflammation. Therefore, lower iron levels in DSS treated *24p3*^−/−^ mice may explain the delayed mortality. In addition, dysregulation of iron metabolism in *24p3*^−/−^ mice especially under a variety of inflammatory conditions has been reported (49).

The results from this study illustrate several potential therapeutic approaches for colitis that are worthy of further investigation. While it may not be feasible to directly induce 24p3, there are clinically feasible approaches that may target 24p3's impact on colitis. For example, recombinant PDGF-BB has seen clinical use for a variety of conditions and our studies suggest that exogenous PDGF-BB may help alleviate symptoms of colitis. In addition, iron chelation may also be another potential therapeutic strategy based upon our studies using iron supplementation.

24p3 is a dual functioning protein in that it plays both proinflammatory and anti-inflammatory roles. Our studies demonstrate a proinflammatory role via previously unreported suppression of PDGF signaling pathway. The connection between 24p3 and PDGF signaling pathway is intriguing and further expands the role of 24p3 besides a regulator of iron metabolism.

## Ethics Statement

This study was carried out in accordance with the recommendations of the Case Western Reserve University IACUC committee. The protocol was approved by the Case Western Reserve University IACUC committee.

## Author Contributions

LRD and DW helped design research studies, analyze data and write the manuscript. ZL, RL, and ND conducted experiments, acquired and analyzed data, and assisted with manuscript preparation. LDM conducted experiments and analyzed data. FC assisted with research design.

### Conflict of Interest Statement

The authors declare that the research was conducted in the absence of any commercial or financial relationships that could be construed as a potential conflict of interest.
